# Laparoscopic vertical sleeve gastrectomy: A 5-year veterans affairs review

**DOI:** 10.1097/MD.0000000000007508

**Published:** 2017-09-01

**Authors:** Rahman G. Barry, Farzad A. Amiri, Todd W. Gress, D. Blaine Nease, Timothy D. Canterbury

**Affiliations:** a Department of Surgery, Marshall University; b Department of Surgery, Huntington Veterans Affairs Medical Center, Huntington, WV.

**Keywords:** bariatric surgery, gastric sleeve, obesity, sleeve gastrectomy, veterans affairs, weight loss

## Abstract

The aim of this study was to evaluate the outcomes after laparoscopic sleeve gastrectomy (SG) in a VA population.

SG has recently gained popularity as a definitive bariatric surgery procedure. Data are lacking on long-term outcomes, particularly in a Veterans Affairs population.

We retrospectively reviewed 223 patients who underwent SG for morbid obesity between January 2009 and June 2014. Data on length of stay, complications, interval weight loss, comorbidities, and number of therapies preoperatively and at long-term follow-up were collected.

There were 164 males and 59 females who underwent SG. The mean body mass index was 45.4 kg/m^2^. Mean excess weight loss at 1 year was 62.9%, and 47.0% at 5 years. Weight loss continued until 12 to 18 months, when there was a nadir in weight loss (*P* < .001). There were 4 deaths and 4 staple-line leaks, with 3 deaths related to late cardiac events. One early death occurred in a very high-risk patient. All staple-line leaks were managed nonoperatively. Of the 223 patients, 193 had hypertension, 137 diabetes, 158 hyperlipidemia, 119 obstructive sleep apnea (OSA), and 125 had gastroesophageal reflux disease. Preoperatively, patients were on a mean of 1.9 antihypertensive and 0.9 hyperlipidemic, anti-reflux and oral hypoglycemic agents. Fifty percent of patients with diabetes were on insulin and 68% with OSA used continuous positive airway pressure/bilevel positive airway pressure (CPAP/BiPAP). We found significant absolute reductions in mean antihypertensive medications (−0.8), hyperlipidemic agents (−0.4), antireflux agents (−0.4), oral hypoglycemics (−0.6), insulin use (−25%), and use of CPAP/BiPAP (−55%) (all *P* < .001).

Laparoscopic sleeve gastrectomy is a safe and effective bariatric surgery procedure, resulting in significant early weight loss up to 18 months and long-term improvement in all major obesity-related comorbid conditions.

## 1 Introduction

Obesity has reached epidemic proportions in the United States and around the world. According to 2014 data from the World Health Organization (WHO), it is estimated that >1.9 billion adults aged 18 years and older were overweight (body mass index [BMI] ≥ 25 kg/m^2^), with >600 million of these considered obese (BMI ≥30 kg/m^2^).^[1]^ The impact of obesity on overall health is significant, with an associated 50% to 100% increased risk of premature death when compared to individuals of a healthy weight.^[2]^ An estimated 300,000 deaths annually are attributed to obesity, with obesity-related comorbid medical conditions contributing substantially to preventable morbidity and mortality.^[3]^

It has been demonstrated that as little as 5% to 15% excess total body weight loss improves obesity-related comorbid conditions, particularly cardiovascular disease in the short term.^[4,5]^ Several studies have demonstrated that significant weight loss results in improvement in type 2 diabetes mellitus (T2DM), hypertension, obstructive sleep apnea (OSA), hyperlipidemia, and gastroesophageal reflux disease (GERD), with the most consistent improvement observed in the setting of bariatric surgery.^[6–11]^

The laparoscopic Roux-en-Y gastric bypass has traditionally been the most widely performed bariatric surgery operation, as well as the criterion standard against which other weight loss operations were measured. There has been a paradigm shift in recent years, with statistics from the American Society for Metabolic and Bariatric Surgery (ASMBS) demonstrating that of the 179,000 bariatric surgery operations performed in 2013, 42.1% were sleeve gastrectomy operations, surpassing the Roux-en-Y gastric bypass, which accounted for 34.2% of bariatric operations.^[12]^

In 1988, Doug Hess performed the first open sleeve gastrectomy operation as part of a 2-stage duodenal switch operation for the super morbidly obese.^[13–14]^ In the late 1990s, it was recognized that there was significant excess weight loss and improvement in obesity-related comorbid conditions even before completion of the second stage of the operation, with outcomes comparable to the 2-stage duodenal switch procedure. It was as a result of these findings that the stand-alone sleeve gastrectomy operation was born, and in 2000, Dr. Michel Gagner performed the first laparoscopic sleeve gastrectomy (SG) as part of a duodenal switch operation, giving rise to the operation performed today.^[15]^

Global acceptance of the SG surgery has been slow. It was not until April 2013 that the American academy of Clinical Endocrinologists, the American Society of Metabolic and Bariatric Surgeons, and The Obesity Society reclassified sleeve gastrectomy as a proven surgical option rather than an investigational one. During the last decade, there has been tremendous enthusiasm to perform this procedure. Given the relatively recent acceptance and practice of this stand-alone operation, data are lacking on long-term outcomes after SG, and this is most notably evident in the Veterans Affairs population.

In 1991, criteria for bariatric surgery eligibility were determined at the National Institutes of Health (NIH) consensus development panel. It was determined that patients should have a BMI of at least 40 kg/m^2^ or a BMI of at least 35 kg/m^2^ with an associated obesity-related comorbid condition.^[16]^ In addition to these requirements, patients should have attempted and failed supervised weight loss and have realistic expectations without recent substance abuse and been evaluated by a dietitian and psychologist preoperatively. Patients should also have no medical contraindications to surgery and be committed to lifelong dietary change and follow-up. Our study population has been particularly affected by obesity. As the impact of obesity and metabolic syndrome in our society becomes more evident, definitions continue to evolve. A recent study identified non-alcoholic fatty liver disease (NAFLD) to have common mechanisms with the development of metabolic syndrome and shares the pathophysiologic basis of insulin resistance, with a significant burden on healthcare costs. The use of abdominal ultrasonography was found to have high specificity for detection of NAFLD and has been suggested to be used as a new criterion to define metabolic syndrome.^[19]^

According to data from the Centers for Disease Control and Prevention Behavioral Risk Factor Surveillance System, the prevalence of obesity for the state of West Virginia has significantly increased during the last 2 decades, and in 2009, the state mean BMI of 31.7 kg/m^2^ was well above the national mean BMI of 27.2 kg/m^2^.^[4]^ The aim of this study is to report our results with SG with up to 5 years of long-term follow-up (LTFU).

## 2 Methods

### 2.1 Study population and preoperative evaluation

We performed a retrospective chart review on 223 patients who underwent SG between January 2009 and June 2014 for morbid obesity at the Veterans Affairs Medical Center (VAMC). All patients met the NIH bariatric surgery eligibility criteria.^[16]^ In addition, they were all required to demonstrate failure to lose weight adequately using conservative measures, including a formal diet plan. Their premorbid obesity-related conditions were clearly defined preoperatively. All patients underwent preoperative evaluation by a dietitian and psychologist. All Surgical Care Improvement Project (SCIP) measures, including perioperative antibiotic administration, were followed. We obtained approval for the study from our institutional review board.

### 2.2 Surgical technique

Surgical technique was consistent in all patients reviewed in this study. All operations were performed by a single, fellowship-trained, bariatric surgeon. All surgeries were performed laparoscopically under general endotracheal anesthesia using a 6-port technique as described by Baltasar.^[17]^ There were no conversions to an open operation. The sleeve was calibrated with a 34-Fr diameter gastrointestinal endoscope. All staple lines were buttressed with seamguards. A leakage test was performed at the time of surgery using high-pressure air insufflation via the endoscope, and on postoperative day 1 via a water-soluble contrast swallow study.

### 2.3 Postoperative course and follow-up

A stage 1 bariatric diet consisting of sugar-free clear liquids was started after the swallow study demonstrates adequate contrast transit without leak. Follow-up was scheduled at 6 weeks via tele-health audiovisual conferencing for patients from neighboring states, and clinic visits for local patients. Subsequent follow-up was scheduled at 3 months, 6 months, and then at 6-month intervals until 2 years postoperatively, after which patients were scheduled annually. Most patients had follow-up through their local Veterans Affairs medical center. Our data represent the collective data from all VA hospitals that assumed care of these patients during their postoperative course.

### 2.4 Data collection

We collected basic demographic information and evaluated the patients’ weights preoperatively, and trended their weights during scheduled postoperative follow-up visits at 6 weeks, 3 months, 6 months, 1 year, 18 months, 2 years, and yearly thereafter until 5 years of follow-up. We reported the following over time according to society standards: weight, BMI, percent excess weight loss (%EWL), and percent excess BMI loss (%EBMIL).^[18]^ We calculated %EWL using ideal weight corresponding to a BMI of 25 kg/m^2^. American Society of Anesthesiologists (ASA) scores were determined based on preoperative Anesthesiologist evaluations.

We assessed obesity-related comorbid conditions preoperatively, including hypertension, T2DM, hyperlipidemia, OSA, and GERD. We documented the number and type of therapies used to control patient comorbid conditions preoperatively, and compared this to the number and type of therapies at LTFU to determine the impact of SG on comorbid conditions. Specifically, we reviewed the number of oral medications required to control hypertension, hyperlipidemia, GERD, and T2DM, in addition to use of insulin for T2DM and continuous positive airway pressure (CPAP) or bilevel positive airway pressure (BiPAP) for OSA. Improvement in comorbid conditions at LTFU was assessed based on decrease in the number of oral medications used for management of T2DM, hypertension, hyperlipidemia, GERD, discontinuation of insulin use for T2DM, and discontinuation of CPAP or BiPAP for OSA. Data were not specifically collected on episodes of reactive hypoglycemia postoperatively; however, on the initial 6-week follow-up visit, there was no documentation of concerning hypoglycemic events occurring postoperatively in diabetic patients. Routine hemoglobin A1c levels were obtained at follow-up in patients with established T2DM. This was followed at subsequent follow-up visits. In most cases, resolution of diabetes was established by normalization of hemoglobin A1c levels when all antidiabetic agents were discontinued, or in rare occasions by fasting glucose measurements and formal oral glucose tolerance tests in accordance with the American Diabetes Association (ADA) guidelines.^[20]^ Patients who presented with established diagnoses of hyperlipidemia were similarly followed with routine lipid panels on all postoperative visits. Resolution of hyperlipidemia was established by normalization of lipid profile values during postoperative follow-up after discontinuation of hyperlipidemic agents per the ACC/AHA guidelines.^[21]^ Our patient pool has long-term data ranging from 1 to 5 years of follow-up depending on the date of the surgery and compliance with follow-up visits.

### 2.5 Statistical analysis

We assessed patient demographics, baseline comorbid conditions, baseline medication use, ASA class, and weight and BMI using simple descriptive statistics. We compared preoperative and postoperative medication use utilizing a paired Student *t* test and confirmed these comparisons using the nonparametric sign rank test for robustness of data. We compared preoperative discontinuation of insulin and CPAP or BiPAP using a proportions test. A 2-tailed *P* value <.05 was considered statistically significant. All analyses were performed using Stata 13.0 (Stata Corp, College Station, TX).

## 3 Results

A total of 223 patients underwent laparoscopic vertical sleeve gastrectomy operations between January 2009 and June 2014. Among these, there were 164 males (74%) and 59 females (26%) with a mean age of 53 years (SD 9.0 years), a mean weight of 139.9 kg (308.1 lbs.) and mean BMI of 45.5 kg/m^2^ (SD 5.2 kg/m^2^). The mean ASA score was 2.6 (SD 0.6) and mean length of stay (LOS) was 4.9 days (SD 3.2; excluded 1 patient with 172-day hospital stay).

Of the 223 patients in our study, the majority had obesity-related comorbid conditions at the time of surgery. There were 193 (87%) patients with hypertension, 137 (61%) with T2DM, 158 (71%) with hyperlipidemia, 119 (53%) with OSA, and 125 (56%) patients with GERD.

**Figure 1:**
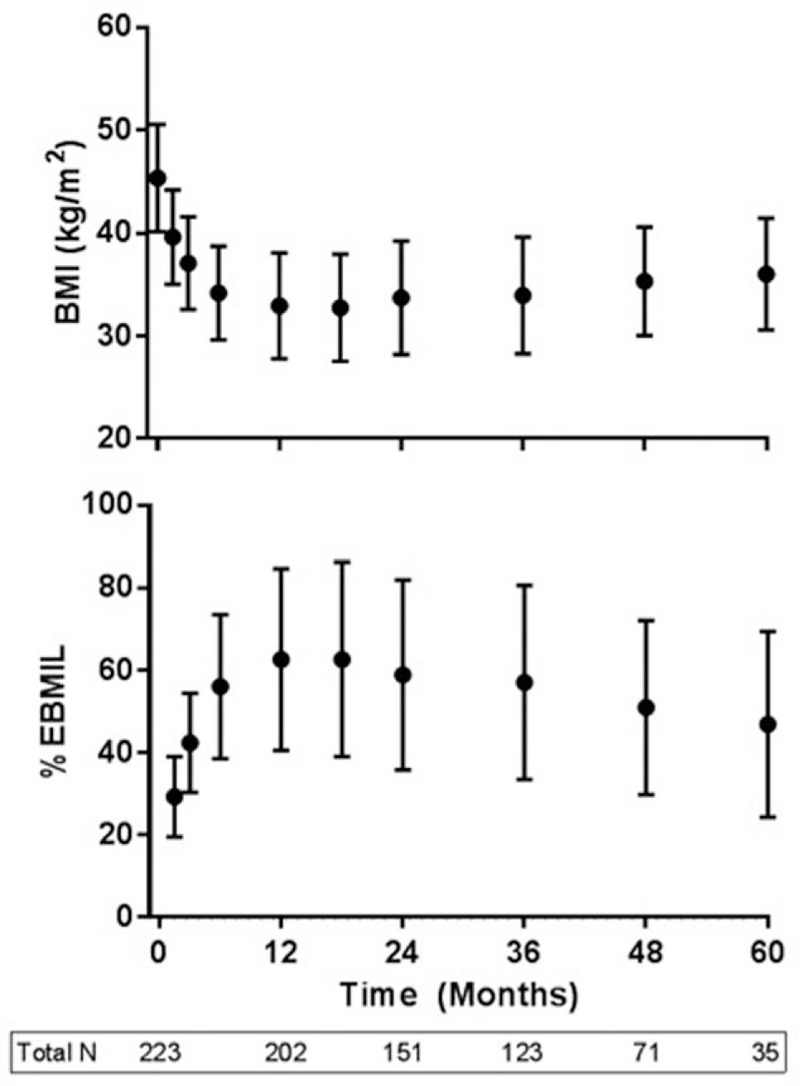
Body mass index and percent excess body mass index loss over time in 223 veteran patients undergoing sleeve gastrectomy. %BMIL = percent body mass index loss. Total N represents number of patients evaluated at time indicated on graph.

**Figure 2:**
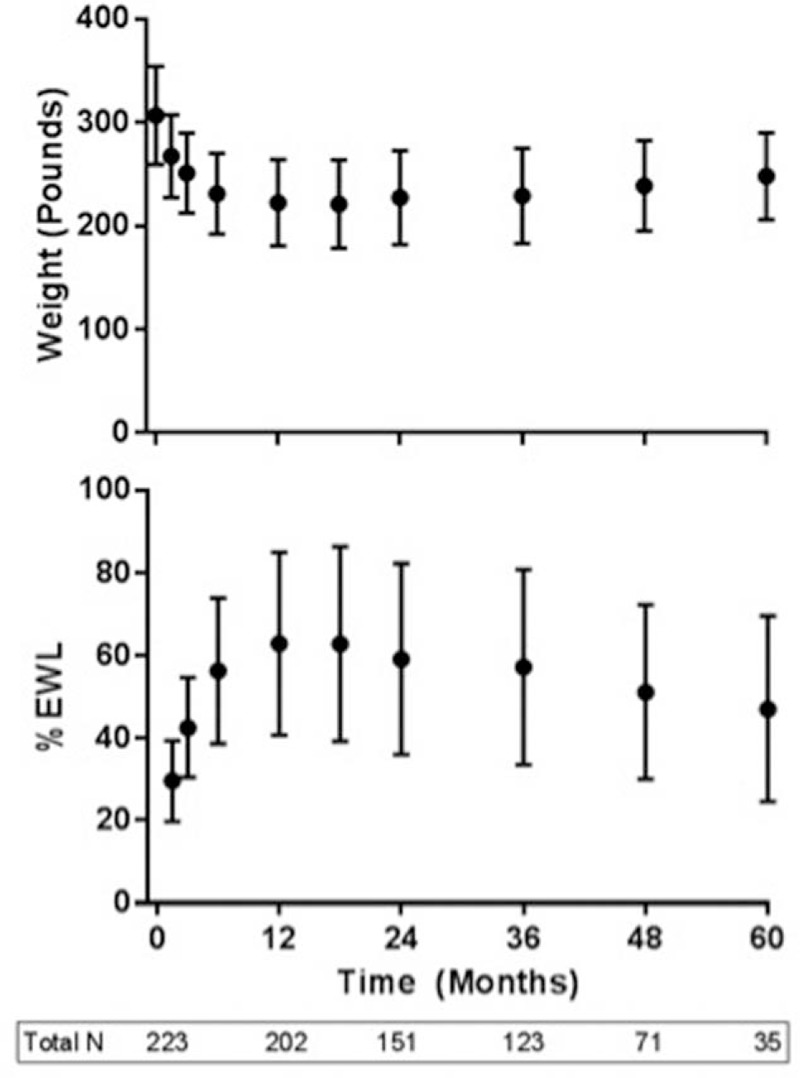
Total body weight and percent excess weight loss over time in 223 veteran patients undergoing sleeve gastrectomy. %EWL = percent excess weight loss. Total N represents number of patients evaluated at time indicated on graph.

The mean preoperative BMI in our study was 45.4 kg/m^2^ with a range from 33.0 to 56.6 kg/m^2^. The percent total body weight loss at 1, 2, and 5 years was 27.8%, 26.2%, and 21.3% respectively. Figures 1 and 2 depict mean BMI and %EBMIL and mean weight and %EWL over time, respectively. The %EBMIL data comply with the new metabolic and bariatric surgery outcome reporting standards established by ASMBS.^[18]^ As can be seen, mean weight and BMI declined rapidly in the initial months following surgery with a more gradual decline to time of maximum weight loss at 12 months. This was followed by a gradual increase in weight and BMI over the ensuing time period. The cohort experienced a peak %EWL of 62.9% at 12 months with a final %EWL of 47.0% at 60 months. At 12 months, women experienced a larger peak %EWL of 67.5% compared to 61.2% for men, but this difference was not statistically significant (*P* = .07).

As can be seen in Table 1, patients with baseline comorbid conditions were on an average of 0.9 to 1.9 medications depending on the condition. Preoperative insulin use for T2DM was at 50.4% and preoperative CPAP or BiPAP use for OSA was at 68.4%. All diabetic patients who were on monotherapy with oral hypoglycemic agents used metformin at varying doses. Among patients on multiple oral hypoglycemic agents, incretin-based therapies were frequently used. All patients had discontinued use of their incretin-based therapies postoperatively. At postoperative assessment, all conditions significantly improved as measured by medication use with mean postoperative medication use decreased to 0.3 to 1 medication depending on the condition (all *P* < .001 for preoperative vs. postoperative medication use). As with mean medication use, insulin use for T2DM went down (postoperative insulin use at 24.8%) and CPAP or BiPAP use for OSA also declined (postoperative CPAP/BiPAP use at 13.7%) (all *P* < .001 for preoperative vs. postoperative use comparison). Of the 34 diabetic patients requiring insulin use postoperatively, 23 patients (67.6%) had a decrease in the dose of insulin required. There were 4 patients who required a dose higher than their preoperative dose at LTFU, and 7 patients who remained on their preoperative insulin dose.

**Table 1:**
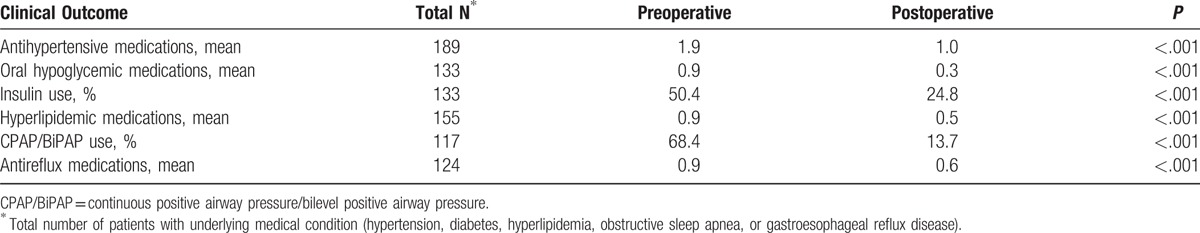
Clinical outcomes measured before and after sleeve gastrectomy.

There were 4 deaths (1.7%) and 4 staple-line leaks (1.7%) in our study population. Three deaths occurred several years postoperatively, and were related to cardiac events. One death occurred 2 months after surgery in a very high-risk patient with multiple comorbidities. This patient had undergone a previous horizontal gastroplasty and had inadequate weight loss. He developed staple-line leak sepsis after his sleeve gastrectomy, and died 2 months later from complications related to his staple-line leak and prolonged hospitalization. All staple-line leaks were managed nonoperatively, with 1 death in this group, as previously mentioned. There were no postoperative deaths at 30 days in our study.

## 4 Discussion

Our study encompasses a unique bariatric population. Of the 156 VA hospitals across the United States, our VAMC has performed, on average, 10% 15% of all VA bariatric surgery operations annually according to the Veterans Affairs Surgical Quality Improvement Program (VASQIP) database. More than 95% of the Bariatric surgery operations at our VAMC are laparoscopic sleeve gastrectomies. With our VAMC being the only regional VA hospital performing frequent bariatric surgery operations, our study population consists of patients outside the local area, including neighboring states. This limits coordination of bariatric follow-up strategies normally implemented in many non-VA institutions nationally.

Our longest follow-up data extended to 5 years postoperatively, and included 35 patients. Of the 223 patients, 213 had at least 1 year of postoperative follow-up data, with a mean LTFU time of 2.5 years. There were 124 patients (56%) with at least 3 years of follow-up data. Although there was a trend toward a higher mean weight and BMI in the female cohort, there were no statistically significant differences in mean weight, BMI, age, excess weight loss, comorbidities, or resolution of comorbid conditions between sexes. We had an approximate 3:1 male to female ratio in our cohort, in addition to a significantly older age group than is seen in most non-VA bariatric populations. This has several implications as it relates to comorbidities and surgical risk. Patients in our cohort tended to have obesity-related comorbidities, which were long-standing, with a tendency to be on a greater number of medications, were more likely to use CPAP or BiPAP to control OSA, and were more likely to be on insulin for T2DM control. There was also a tendency to have a greater number of cardiovascular risk factors, a higher ASA class, and a more sedentary lifestyle than in a non-VA bariatric surgery patient cohort. The complexity of care was further increased by the fact that postoperative follow-up and care had to be coordinated at a distance, between cities and states. Initial 6-week, 3-month, 6-month, and 1-year visits were conducted via Telehealth audiovisual conferencing, with subsequent care coordinated through their local VA hospitals. This was facilitated by the VA electronic medical record system, which allows access to all VA health records across the United States.

Analysis of the interval weight loss data demonstrates that there was significant early weight loss seen universally in all patients reviewed. This persisted until 12 to 18 months, when there was a nadir in weight loss and a subsequent tendency to regain weight. It has been well described that the gastric sleeve tends to become more compliant with time, essentially becoming less “restrictive.” This likely contributed significantly to the increase in weight seen after 12 to 18 months in our study. The increased sedentary nature of our cohort and the lack of standardized follow-up protocols with ongoing dietary and exercise counseling likely also contributed significantly to this outcome.

Evaluation of outcomes at LTFU demonstrates that SG is a safe and effective surgery for weight loss when the appropriate indications are met, with significant early weight loss, and a zero 30-day mortality despite the increased surgical risk in our population. Despite the tendency to regain weight beyond 18 months, there was still significant excess weight loss that persisted at LTFU, with 47.0% EWL at 5 years. There were also statistically significant improvements in all obesity-related comorbid conditions that persisted long term. Although there was an overall improvement in GERD, there were 29 new cases requiring antireflux agents. This highlights the refluxogenic nature of a sleeve gastrectomy operation, but still demonstrates that there is an overall benefit in patients with GERD. The average hospital LOS was 4.9 days compared to previously reported averages of 3 days. Because we are a regional VA medical center offering bariatric surgery to surrounding states, patients tended to have longer hospital stays through weekends and until they were comfortable to return to their home state.

Resolution of obesity-related comorbid conditions was assessed based on a decrease in the number of therapies needed to treat their medical conditions based on physician evaluation. Obesity-related comorbidities being managed with lifestyle modifications were not included in this study, and changes in the dose of individual medications at LTFU were also not evaluated. This would suggest that improvement in comorbid conditions was likely significantly underestimated in this study. The outcomes in our study population were likely significantly affected by the lack of effective follow-up. It would appear that the effectiveness of a SG surgery decreases after 12 to 18 months, in part related to increased compliance of the gastric sleeve, and loss of the restrictive component of this operation. It therefore behooves the provider to ensure that appropriate patient counseling is conducted, and that measures be implemented to ensure effective follow-up, particularly as it relates to dietary and exercise commitments, which become increasingly important after 18 months to ensure continued and sustained weight loss. Based on the findings from this study, patients who undergo a SG operation will likely benefit from an 18-month follow-up visit, in addition to their routine follow-up that focuses on dietary and exercise strategies and further patient education to improve overall weight loss outcomes. This is particularly applicable to institutions where standardized follow-up protocols are not established.

## 5 Conclusion

The benefits to be obtained from laparoscopic vertical sleeve gastrectomy operations are apparent. Our retrospective review of a VA bariatric population demonstrates that SG can be performed safely with a low morbidity and mortality rate. Our cohort achieved substantial weight loss up to 18 months, with sustained weight loss up to 5 years postoperatively despite a tendency to regain weight after 18 months. We demonstrated statistically significant long-term improvement in all major obesity-related comorbid conditions, including T2DM, hypertension, OSA, hyperlipidemia, and GERD. With favorable outcomes and a low overall risk, the SG proves to be a valuable primary treatment modality for morbid obesity in this population, and justifies consideration as the first-line surgical treatment of morbid obesity over the Roux-en-Y gastric bypass.
